# Endocrine and neuromodulatory regulation during prolonged maximal 6000-m rowing in male athletes

**DOI:** 10.1007/s00421-026-06325-3

**Published:** 2026-06-29

**Authors:** Joanna Ostapiuk-Karolczuk, Anna Kasperska, Hanna Dziewiecka, Justyna Cichoń-Woźniak, Małgorzata Reysner, Wojciech Gruszka, Piotr Basta, Sabina Kaczmarczyk, Anna Skarpańska-Stejnborn

**Affiliations:** 1 Department of Biological Sciences, Faculty of Sport Sciences in Gorzow Wielkopolski, Poznan University of Physical Education, Estkowskiego 13, 66-400 Gorzow Wielkopolski, Poland; 2 Department of Palliative Medicine, University of Medical Sciences, 61-701 Poznan, Poland; 3 Department of Physical Education and Sports, Faculty of Sport Sciences in Gorzow Wielkopolski, Poznan University of Physical Education, Estkowskiego 13, 66-400 Gorzow Wielkopolski, Poland

**Keywords:** Prolonged maximal exercise, Rowing ergometer, Peripheral monoamines, Endocrine response, Early recovery

## Abstract

**Purpose:**

The 6000-m rowing ergometer test represents a prolonged maximal effort commonly used in elite rowing; however, its physiological responses are less well characterized than those observed during the standard 2000-m race-distance format. This study aimed to examine acute endocrine and neuromodulatory responses to a maximal 6000-m rowing test in elite male rowers.

**Methods:**

Nineteen elite male rowers performed a maximal 6000-m rowing ergometer test. Venous blood samples were collected before exercise (PRE), immediately after exercise (POST), and after 1 h of recovery (1-h REC). Serum concentrations of cortisol, testosterone, serotonin, dopamine, β-endorphin, anandamide, and 2-arachidonoylglycerol were determined. Testosterone-to-cortisol and serotonin-to-dopamine ratios were calculated. Achievement motivation was assessed before exercise to describe task engagement.

**Results:**

Cortisol concentrations did not change across time points. Testosterone decreased at POST and increased above baseline at 1-h REC, resulting in corresponding changes in the testosterone-to-cortisol ratio. Serotonin concentrations decreased after exercise and remained reduced during recovery, whereas dopamine increased at POST and remained elevated at 1-h REC, leading to a reduced serotonin-to-dopamine ratio. β-Endorphin increased after exercise and remained elevated during recovery. Anandamide concentrations did not change significantly, while 2-arachidonoylglycerol showed a transient post-exercise decrease. Achievement motivation was not associated with acute physiological responses.

**Conclusion:**

Prolonged maximal rowing induced a physiological response characterized by distinct temporal patterns of endocrine and peripheral neuromodulatory markers during early recovery. These responses differ from those typically observed during race-distance testing and may contribute to the characterization of early post-exercise responses following prolonged maximal rowing exercise.

## Introduction

Rowing is a metabolically demanding endurance sport that requires the integration of aerobic capacity, technical efficiency, and physiological stress regulation. Accordingly, laboratory-based rowing ergometer tests are routinely used to assess training status in elite rowers, with the majority of available evidence derived from the standard 2000-m race-distance protocol lasting approximately 6–7 min (Mikulić 2009). Research using this short, high-intensity format has substantially advanced understanding of acute endocrine and metabolic responses in rowing, including activation of the hypothalamic-pituitary-adrenal axis, transient reductions in the testosterone-to-cortisol ratio, and pronounced post-exercise elevations in blood lactate concentration (Mäestu et al. 2005; Cadegiani and Kater 2017).

In contrast, longer rowing ergometer tests remain less well characterized despite their widespread use in elite training practice. The 6000-m rowing ergometer test represents a prolonged maximal effort lasting approximately 20–24 min and engaging exercise within the heavy-to-severe intensity domain (Mikulić 2009). Compared with the 2000-m protocol, the 6000-m test involves sustained metabolic strain, progressive fatigue accumulation, and reduced reliance on maximal anaerobic energy turnover, thereby increasing the importance of pacing strategies and systemic regulatory mechanisms. Despite its applied relevance, the physiological characteristics of this prolonged rowing-specific maximal task remain incompletely described (Meeusen et al. 2006; Pachciarek et al. 2025; Raichlen et al. 2013).

However, it remains unclear whether prolonged maximal rowing elicits coordinated or dissociated patterns of endocrine, neuromodulatory, and endocannabinoid responses that differ from those described for the standard 2000-m race-distance protocol.

Regulation of performance during prolonged high-intensity exercise depends on coordinated endocrine responses supporting metabolic homeostasis and stress adaptation. Cortisol secretion typically increases in relation to exercise intensity and duration, while testosterone dynamics reflect acute changes in anabolic-catabolic balance (Hackney 2020). Although these hormonal responses are well documented during short maximal exercise, their integrated behavior during prolonged maximal rowing efforts remains insufficiently defined.

Beyond classical endocrine regulation, neuromodulatory systems play an important role in effort perception, motivation, and fatigue tolerance during sustained exercise. Alterations in serotonin- and dopamine-related pathways have been discussed in the context of exercise-induced physiological regulation and pacing during prolonged high-intensity tasks. (Meeusen et al. 2006; Roelands and Meeusen 2010). In addition, β-endorphin release reflects nociceptive modulation during strenuous endurance exercise, whereas endocannabinoids such as anandamide and 2-arachidonoylglycerol contribute to affective regulation and stress buffering in an intensity-dependent manner (Sparling et al. 2003; Raichlen et al. 2013). Despite extensive research in endurance sports, these regulatory systems have rarely been examined simultaneously within a prolonged rowing-specific context.

Psychological characteristics may further influence how athletes approach prolonged maximal tasks. Pre-exercise psychological state and achievement motivation can affect task engagement and effort regulation, particularly when external pacing cues are limited (Beedie et al. 2000; Lochbaum et al. 2021). In the present study, achievement motivation was assessed before exercise to characterize task engagement during a prolonged maximal rowing effort rather than to predict acute physiological responses.

Therefore, this study aimed to characterize acute endocrine (cortisol, testosterone), neuromodulatory (serotonin, dopamine, β-endorphin), and endocannabinoid (anandamide, 2-arachidonoylglycerol) responses to a maximal 6000-m rowing ergometer test in elite male rowers, and to assess pre-exercise achievement motivation to characterize task engagement before this prolonged maximal effort.

## Materials and methods

### Ethics approval

This observational study was approved by the Bioethical Committee of the Poznan University of Medical Sciences (decision no. 685/23, 2023) and conducted in accordance with the Declaration of Helsinki. The study was registered retrospectively at ClinicalTrials.gov (NCT07235124; 14 November 2025) due to its observational design and the absence of any intervention affecting participant safety, to ensure transparency and compliance with reporting standards. All participants were informed about the study procedures and potential risks and provided written informed consent before participation. Participation was voluntary, and each athlete had the right to withdraw from the study at any time without giving a reason.

### Study participants

Nineteen elite male rowers, all active members of the Polish National Rowing Team with at least five years of systematic rowing training and regular participation in national and international championship competitions, participated in this study. The cohort was homogeneous in terms of sex, sport discipline, competitive level, training background, recent training exposure, and testing environment. All athletes followed the same centralized training program, with standardized accommodation and nutrition during the training camp. All were medically fit, without any acute or chronic health problems. Inclusion criteria required that all participants had at least five years of rowing training, were active members of the national rowing team, completed the 6000-m rowing ergometer test according to the study protocol, and completed the Achievement Motivation Inventory (LMI).

Exclusion criteria included any signs of acute or chronic inflammation, musculoskeletal pain or injury that could affect rowing performance, the use of anti-inflammatory or hormonal medications, and any failure to follow the testing procedures or provide complete data.

Anthropometric measures included body mass, body composition (% fat, fat-free mass) assessed using a bioelectrical impedance analyzer (Tanita BC-418; Tanita Corporation, Tokyo, Japan), and body height measured with a stadiometer (SECA 213; seca GmbH & Co. KG, Hamburg, Germany). Measurements were taken in the morning, after an overnight fast (Table 1).

**Table 1:** Participant characteristics

	Male *N*=19
Age (years)	19.43 ± 1.09
Body mass (kg)	84.11 ± 8.34
Height (cm)	186.6 ± 5.32
Fat (%)	10.55 ± 3.09
Fat–free Mass (kg)	75.08 ± 6.00

### Study design

The study was conducted in November, during a period of reduced training load following the competitive season. This timing was selected to minimize the influence of acute competition-related fatigue while still reflecting the athletes’ regular training environment. The training program and training load during the 7 days preceding the 6000-m rowing test are presented in Table 2. The training structure during the 7 days preceding the test was recorded to characterize recent training exposure and to support interpretation of baseline hormonal concentrations. All athletes participated in a centralized training camp during this period, ensuring standardized training, living, and nutritional conditions, thereby minimizing environmental variability. Participants were instructed to avoid caffeine-containing beverages and supplements for at least 12 h before testing and to refrain from strenuous physical activity outside the standardized training program during the 24 h preceding the exercise test. Athletes were also instructed to maintain their habitual hydration practices before testing.

**Table 2:** Training program for 7 days before the rowing test

Training structure	Days before the 6000 m exercise test						
	1	2	3	4	5	6	7
Total training time, min/day	110	220	110	220	100	190	80
Time rowed, min/day	60	40	70	80	80	20	–
Distance rowed, km/day	12	8	12	20	20	4	–
Training for force development, min/day	–	90	–	100	–	100	–
Extensive endurance rowing training time, min/day	42	40	20	80	50	20	–
High intensity endurance rowing training time, min/day	18	–	50	–	30	–	–
Unspecific training (running, etc.), min/day	50	90	40	40	20	90	80

On the testing day, venous blood was first collected in a resting, fasted state to obtain baseline values (PRE) between 07:00 and 09:00 to minimize circadian variability. After baseline blood collection, participants completed the Achievement Motivation Inventory to assess pre-exercise motivational characteristics. Subsequently, athletes performed a 5-min individualized warm-up, followed by a maximal 6000-m rowing ergometer test on a Concept 2 ergometer (Concept 2 Inc., USA). The 6000-m protocol is commonly used in high-performance rowing as a valid measure of aerobic capacity and endurance performance in elite athletes (Mikulic, 2009). Because the test served as a qualification criterion for the World Championship selection, it was performed under conditions of high competitive motivation, with standardized verbal encouragement provided throughout the test to ensure maximal effort.

Venous blood samples were collected immediately after completion of the exercise bout (POST) and after 1 h of recovery (1 H REC) from the antecubital vein by an experienced registered nurse using standardized sterile procedures, collected into serum tubes with a clot activator (Sarstedt AG & Co., Germany), allowed to clot for approximately 30 min, centrifuged at 3000 rpm for 10 min, aliquoted, and stored at −80 °C until analysis. Total time to complete the 6000-m distance and mean power output were recorded as primary performance outcomes (Table 3). All procedures were conducted under standardized laboratory conditions by trained personnel to minimize pre-analytical variability.

**Table 3:** Results of the rowing ergometer test over a 6000-m distance

	Mean ± SD
LA pre-exercise (mmol·L⁻¹)	1.96 ± 0.40
LA post-exercise (mmol·L⁻¹)	10.29 ± 3.40
Exercise time (s)	1250.00 ± 38.93
Power (W)	313.68 ± 34.80
Relative power (W·kg⁻¹)	3.75 ± 0.50

Serum concentrations of testosterone and cortisol were measured using enzyme-linked immunosorbent assay (ELISA) kits (DiaMetra, Spello, Italy) according to the manufacturer’s instructions. For testosterone, the analytical sensitivity was 0.10 ng·mL⁻¹, the assay range was 0.10–14.0 ng·mL⁻¹, and the intra- and inter-assay coefficients of variation were ≤ 7.0 and ≤ 8.3%, respectively. For cortisol, the analytical sensitivity was 2.42 ng·mL⁻¹, and the intra- and inter-assay coefficients of variation were ≤ 5.1 and ≤ 11.0%, respectively. The manufacturer did not specify an assay range for the cortisol kit. Serum concentrations of serotonin, dopamine, β-endorphin (β-EP), anandamide (AEA), and 2-arachidonoylglycerol (2-AG) were determined using commercially available ELISA kits (Shanghai Sunred Biological Technology Co., Ltd., China) according to the manufacturer’s instructions. Analytical sensitivities and assay ranges were 0.388 ng·L⁻¹ and 0.5–120 ng·L⁻¹ for serotonin, 7.043 nmol·L⁻¹ and 8–2000 nmol·L⁻¹ for dopamine, 2.451 ng·mL⁻¹ and 3–600 ng·L⁻¹ for β-endorphin, 7.125 ng·mL⁻¹ and 8–2000 ng·mL⁻¹ AEA, and 0.105 ng·L⁻¹ and 0.2–30 ng·L⁻¹ for 2-AG, respectively. Intra-assay and inter-assay coefficients of variation were < 10 and < 12%, respectively for these kits. All measurements were performed using a SPECTROstar Nano microplate reader (BMG Labtech, Germany). Dopamine concentrations were converted to ng·mL⁻¹ to allow consistency in data presentation. The testosterone-to-cortisol (T/C) ratio was calculated as a supportive index of anabolic-catabolic balance, while the serotonin-to-dopamine (S/D) ratio was calculated to describe the relative pattern of peripheral monoaminergic responses. Lactate concentration (LA) was assessed in capillary blood samples collected immediately after exercise using the Lactat Photometer and a commercially available diagnostic kit (Diaglobal GmbH, Berlin, Germany), according to the manufacturer’s instructions.

### Psychological assessment of LMI

To assess the athletes’ motivational characteristics before the exercise test, the Achievement Motivation Inventory was administered in the Polish adaptation by Klinkosz and Sękowski (2013). The LMI is a standardized and psychometrically validated instrument designed to measure achievement-related motivation across multiple dimensions. In the present study, three key motivational components were analyzed: self-confidence, ambition, and self-control. The questionnaire was administered in its validated paper-and-pencil format under standardized conditions, immediately before the exercise test. All participants completed the inventory individually, in a quiet and controlled environment, under the supervision of a trained psychologist.

### Statistical analysis

Statistical analyses were conducted using Statistica 14.0 (TIBCO Software Inc., Palo Alto, CA, USA), while graphical data presentation was performed using GraphPad Prism 10.0 (GraphPad Software Inc., San Diego, CA, USA). Descriptive statistics are shown as mean ± standard deviation (SD). Scores for the three components of the LMI questionnaire (Self-Confidence, Ambition, Self-Control) were summarized descriptively, as they were collected only once before the exercise test.

An a priori power analysis for a within-subject repeated-measures ANOVA with three time points was conducted using G*Power 3.1 (Heinrich Heine University Düsseldorf, Düsseldorf, Germany). Based on prespecified parameters (medium effect size, f = 0.25; α = 0.05; statistical power = 0.80; inter-measure correlation = 0.50), the minimum required sample size was estimated at 18 participants. The final sample consisted of 19 athletes.

Normality of data distribution was assessed using the Shapiro-Wilk test. Sphericity was evaluated using Mauchly’s test, and when violated, the Greenhouse-Geisser correction was applied. Among the analyzed variables, only 2-arachidonoylglycerol (2-AG) deviated from normal distribution and was log-transformed before analysis; all results are reported in original units for ease of interpretation. Changes in biochemical variables across PRE, POST, and 1-h recovery were analyzed using repeated-measures ANOVA. When a significant main effect of time was observed, Bonferroni-adjusted post hoc comparisons were applied to reduce the risk of inflated type I error associated with multiple comparisons.

Effect sizes were reported to quantify the magnitude of observed effects. Partial eta squared (η²ₚₚ) was calculated for ANOVA models and interpreted as small (≥ 0.01), medium (≥ 0.06), or large (≥ 0.14). For pairwise comparisons, Cohen’s d for repeated measures was calculated and interpreted as trivial (< 0.20), small (0.20–0.49), moderate (0.50–0.79), or large (≥ 0.80), according to established conventions (Cohen 1988; Richardson 2011).

Exploratory Pearson correlation analyses were performed for post-exercise biochemical markers to examine relationships among endocrine, neuromodulatory, and endocannabinoid variables. Correlation coefficients were interpreted as small (0.10–0.29), moderate (0.30–0.49), or large (≥ 0.50) (Cohen 1988; Hemphill 2003). Correlation matrices were visualized using heat maps.

All statistical tests were two-tailed, with statistical significance set at *p* < 0.05. No missing data was observed, and all participants completed the full testing protocol.

## Results

**Fig. 1:**
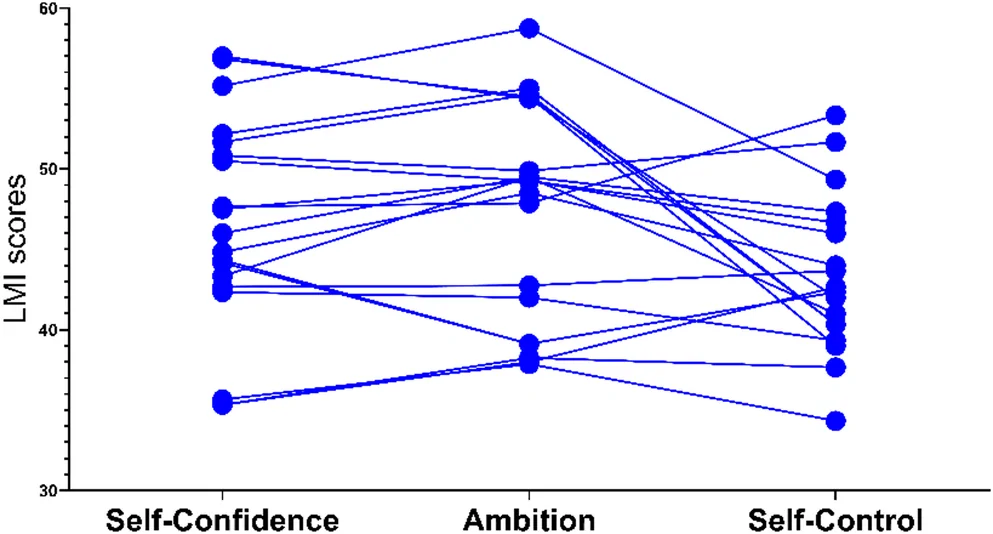
Scores for the three components of the LMI questionnaire (Self-Confidence, Ambition, and Self-Control) before the 6000-m rowing ergometer test.

*Motivation scores***.** The three LMI components showed distinct score levels (Fig. 1). Self-Confidence and Ambition had the highest mean values and showed the largest variability across athletes. In contrast, Self-Control clearly had a lower mean score and a narrower distribution. These data describe the motivation components assessed before the 6000-m rowing ergometer test.

Values are mean ± SD.

**Fig. 2:**
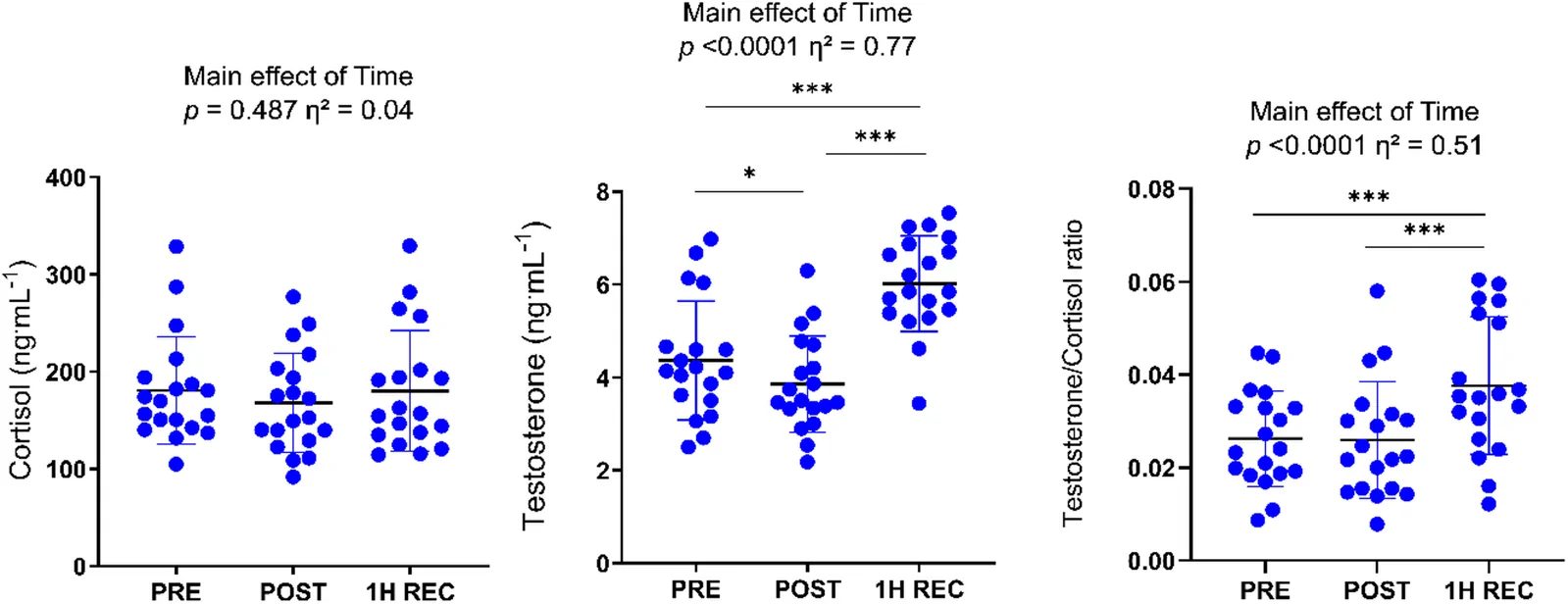
Cortisol, Testosterone, and Testosterone/Cortisol ratio in response to a 6000-m rowing ergometer test.

*Hormonal responses*. There was no main effect of Time for cortisol (*p* = 0.487, η²ₚ = 0.04). Cortisol concentrations showed no statistically significant time-dependent variation across PRE, POST, and 1 H REC. Effect sizes showed small changes (PRE→POST: *d* = 0.21; PRE→1 H REC: *d* = 0.0008; POST→1 H REC: *d* = 0.23) (Fig. 2).

A significant main effect of Time for testosterone was observed (*p* < 0.0001, η²ₚ = 0.77). Testosterone significantly decreased after exercise (*p* < 0.05) and significantly increased after 1 h of recovery (*p* < 0.001). Effect sizes showed large changes (PRE→POST: *d* = 0.58; PRE→1 H REC: *d* = 1.57; POST→1 H REC: *d* = 2.85) (Fig. 2).

There was a significant main effect of Time for the T/C ratio (*p* < 0.0001, η²ₚ = 0.51). The T/C ratio remained stable from PRE to POST but increased substantially at 1 H REC (*p* < 0.001). Corresponding effect sizes ranged from trivial to large (PRE→POST: *d* = 0.033; PRE→1 H REC: *d* = 1.63; POST→1 H REC: *d* = 1.06) (Fig. 2).

Values are mean ± SD. PRE - before exercise; POST - immediately after exercise; 1 H REC − 1 h of recovery. *p < 0.05 **p < 0.01 *** *p* < 0.0001.

**Fig. 3:**
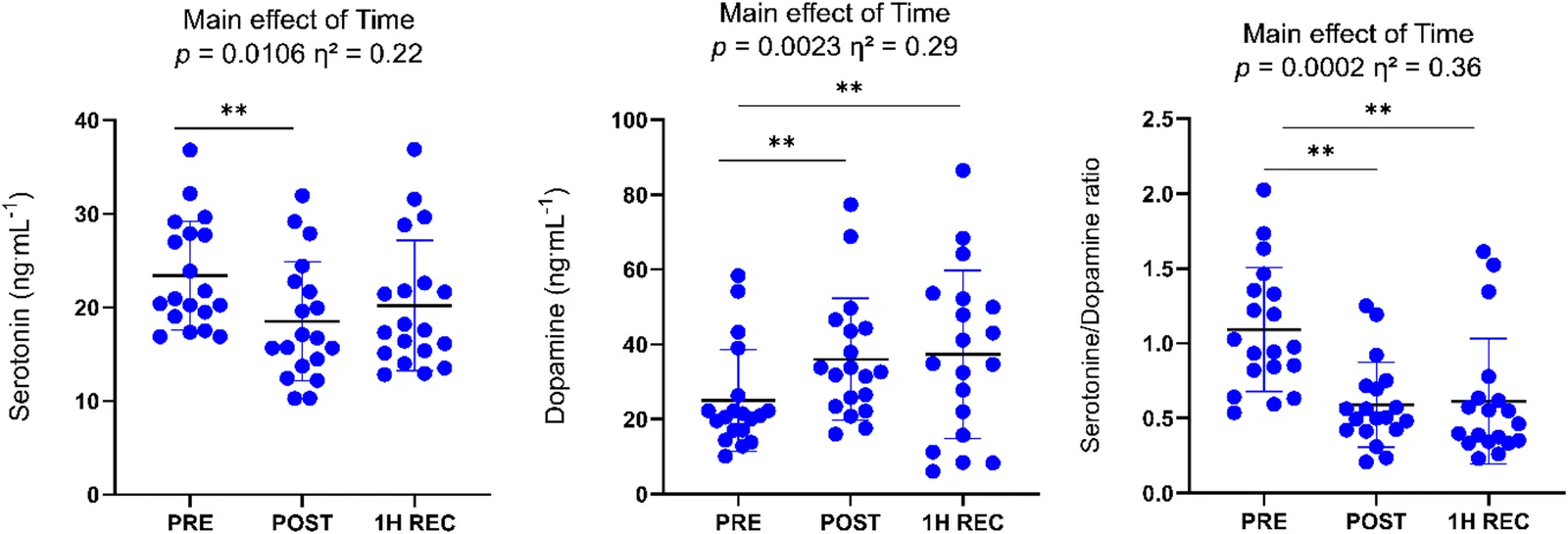
Serotonin, Dopamine, and Serotonin/Dopamine ratio in response to a 6000-m rowing ergometer test

*Serotonin, dopamine, and S/D ratio*. There was a significant main effect of Time for serotonin (*p* = 0.0106, η²ₚ = 0.22). Serotonin was significantly lower POST compared with PRE (*p* < 0.05) and remained reduced at 1 H REC (*p* > 0.05). Effect sizes showed small-to-moderate changes (PRE→POST: *d* = 0.64; PRE→1 H REC: *d* = 0.49; POST→1 H REC: *d* = 0.28) (Fig. 3).

Values are mean ± SD. PRE - before exercise; POST - immediately after exercise; 1 H REC − 1 h of recovery. *p < 0.05 **p < 0.01 *** *p* < 0.0001.

There was a significant main effect of Time for dopamine (*p* = 0.00238, η²ₚ = 0.29). Dopamine was significantly higher POST compared with PRE (*p* < 0.01) and remained significantly elevated at 1 H REC (*p* < 0.05). Effect sizes were moderate to large (PRE→POST: *d* = 0.93; PRE→1 H REC: *d* = 0.77; POST→1 H REC: *d* = 0.02) (Fig. 3).

There was a significant main effect of Time for the S/D ratio (*p* = 0.00029, η²ₚ = 0.36). The ratio was significantly lower POST compared with PRE (*p* < 0.01) and remained significantly reduced at 1 H REC (*p* < 0.05). Effect sizes were large (PRE→POST: *d* = 1.07; PRE→1 H REC: *d* = 1.03; POST→1 H REC: *d* = 0.11) (Fig. 3).

**Fig. 4:**
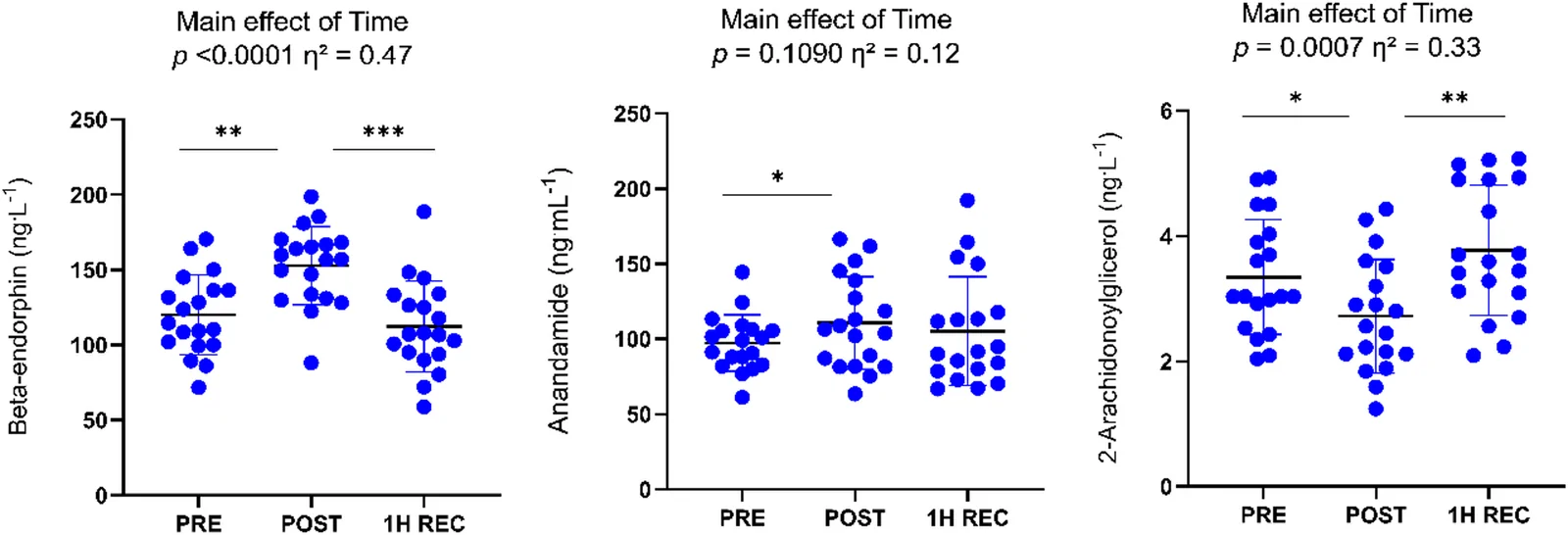
β-Endorphin, anandamide (AEA), and 2-arachidonoylglycerol (2-AG) in response to a 6000-m rowing ergometer test

*β-endorphin and endocannabinoids*. There was a significant main effect of Time for β-endorphin (*p* < 0.0001, η²ₚ = 0.47). β-endorphin was significantly higher POST compared with PRE (*p* < 0.01) and remained significantly higher at 1 H REC (*p* < 0.05). Effect sizes showed large changes (PRE→POST: *d* = 1.02; PRE→1 H REC: *d* = 0.26; POST→1 H REC: *d* = 1.04) (Fig. 4).

 Values are mean ± SD. PRE - before exercise; POST - immediately after exercise; 1 H REC − 1 h of recovery. *p < 0.05 **p < 0.01 *** *p* < 0.0001.

There was no significant main effect of Time for AEA (*p* = 0.109, η²ₚ = 0.12). AEA concentrations increased significantly from PRE to POST (p<0.05), and returned to baselilne after 1 H REC. Effect sizes were small (PRE→POST: *d* = 0.61; PRE→1 H REC: *d* = 0.38; POST→1 H REC: *d* = 0.92) (Fig. 4).

There was a significant main effect of Time for 2-AG (*p* = 0.00071, η²ₚ = 0.33). 2-AG was significantly lower POST than PRE (*p* < 0.05) and significantly higher at 1 H REC than POST (*p* < 0.01). Effect sizes showed moderate-to-large changes (PRE→POST: *d* = 0.61; PRE→1 H REC: *d* = 0.38; POST→1 H REC: *d* = 0.92) (Fig. 4).

**Fig. 5:**
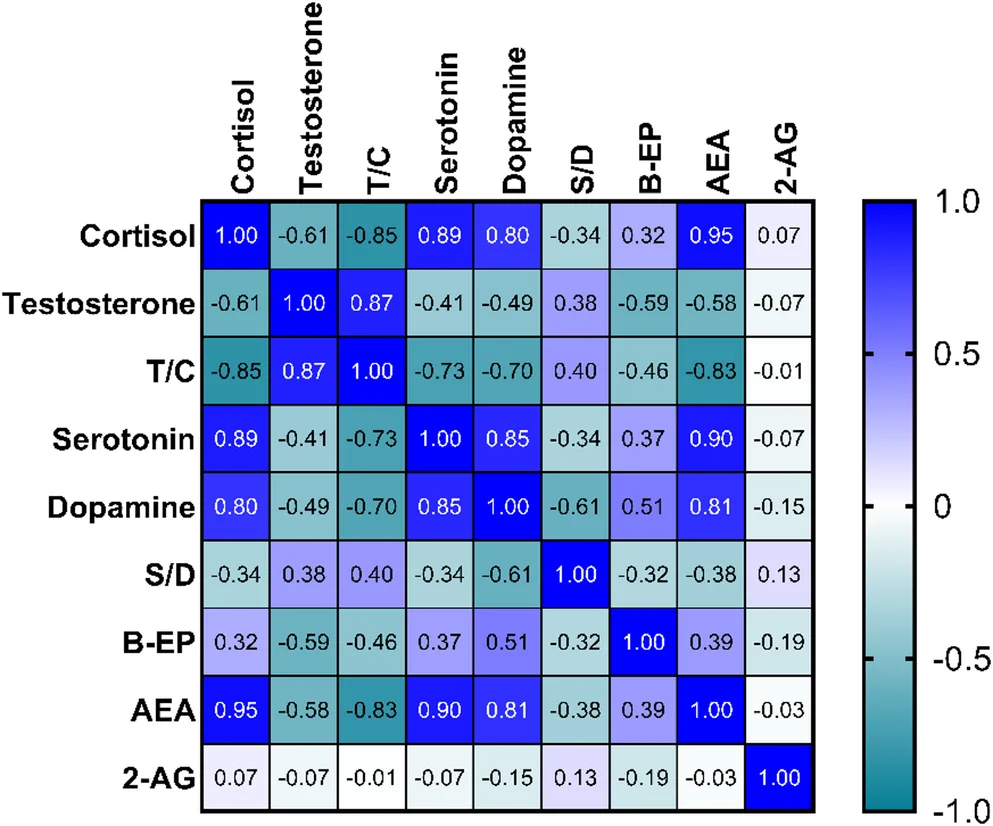
Correlation matrix of post-exercise hormonal, neuromodulatory, and endocannabinoid markers

*Correlation matrix of biochemical markers*. Exploratory correlation analysis of post-exercise biochemical markers revealed primarily domain-specific relationships (Fig. 5). A strong positive correlation was observed between testosterone and the testosterone-to-cortisol ratio (*r* = 0.872, *p* < 0.0001), reflecting their mathematical and physiological interdependence. Within the neuromodulatory domain, serotonin and dopamine were strongly correlated (*r* = 0.846, *p* < 0.0001). In addition, serotonin showed a strong positive association with anandamide (AEA; *r* = 0.901, *p* < 0.0001), while dopamine was also positively correlated with AEA (*r* = 0.814, *p* < 0.0001).

*T/C* testosterone-to-cortisol ratio, *S/D* serotonin-to-dopamine ratio, *β-EP* β-endorphin; *AEA* anandamide, *2-AG* 2-arachidonoylglycerol.

In contrast, correlations between endocrine, neuromodulatory, and endocannabinoid markers were generally weak or inconsistent. Notably, 2-arachidonoylglycerol (2-AG) did not exhibit meaningful correlations with the other biochemical variables. Given the exploratory nature of these analyses, the homogeneous sample, and the limited sample size, these correlation findings should be interpreted descriptively rather than as evidence of functional coupling between regulatory systems.

## Discussion

The present study examined acute endocrine, neuromodulatory, opioid, and endocannabinoid responses to a prolonged maximal 6000-m rowing ergometer test in elite male rowers. In contrast to the extensively studied 2000-m race-distance format, this prolonged maximal effort produced a distinct pattern of exercise-induced physiological responses characterized by stable cortisol concentrations, biphasic testosterone dynamics, divergent peripheral monoaminergic responses, pronounced β-endorphin activation, and limited endocannabinoid involvement. These findings indicate that prolonged maximal rowing is characterized by physiological response patterns that differ from those typically observed during standard race-distance efforts.

The novel contribution of this study lies in the simultaneous assessment of endocrine, peripheral monoaminergic, opioid, and endocannabinoid responses to a prolonged maximal rowing task. Cortisol concentrations did not change following the 6000-m test, despite the maximal nature of the effort. This response contrasts with the consistent post-exercise cortisol elevations reported after 2000-m rowing tests and other short, high-intensity exercise protocols (Mäestu et al. 2005; Ostapiuk-Karolczuk et al. 2025). Attenuated post-exercise cortisol responses have previously been observed in endurance-trained athletes and may reflect cumulative training load, altered hypothalamic-pituitary-adrenal axis responsiveness, or differences in secretion timing during prolonged exercise (Hill et al. 2008; Duclos et al. 2003). Importantly, cortisol during prolonged exercise may peak during the effort rather than immediately after cessation, and the absence of a post-exercise increase should therefore not be interpreted as reduced physiological strain. Overall, cortisol responses to prolonged maximal rowing appear to differ primarily in timing and expression from those observed during shorter maximal protocols.

Baseline testosterone concentrations were within the expected physiological range for adult males and should be interpreted in the context of endurance-trained athletes, in whom lower resting testosterone concentrations have been frequently reported (Hackney 2008; Lane & Hackney, 2015). In response to the 6000-m test, testosterone decreased transiently immediately after exercise, then recovered above baseline at 1 h. Similar biphasic patterns have been reported following strenuous endurance exercise and are attributed to acute autonomic and metabolic influences on hypothalamic-pituitary-gonadal axis regulation (Dote-Montero et al. 2021; Vaamonde et al. 2022). The rapid normalization and rebound observed in the present study suggest a transient endocrine response rather than sustained alteration of the anabolic–catabolic profile. In parallel, the testosterone-to-cortisol ratio showed a transient disturbance after exercise and a marked increase during early recovery, suggesting a short-lived change in the anabolic–catabolic profile rather than prolonged endocrine dysregulation (Crewther et al. 2009; De Luccia et al. 2018). Together, these findings indicate marked temporal changes in endocrine markers during early recovery following prolonged maximal rowing in elite athletes.

A key observation of the present study was the divergent peripheral monoaminergic response pattern. Serotonin concentrations decreased immediately after exercise and remained reduced during early recovery, whereas dopamine increased and remained elevated. Previous studies have reported heterogeneous serotonin responses to endurance exercise, influenced by exercise duration, intensity, and sampling timing (Meeusen et al. 2006; Cordeiro et al. 2017; Zimmer et al. 2018). Sustained post-exercise dopamine elevation has been observed in trained athletes performing high-intensity tasks and may reflect a prolonged peripheral dopaminergic response persisting during the recovery period (Chaouloff 1997; Cordeiro et al. 2017). Together, these findings indicate sustained peripheral monoaminergic alterations during early recovery following prolonged maximal rowing exercise.

The reduced serotonin-to-dopamine ratio observed after the 6000-m test describes the relative shift in peripheral monoaminergic markers during early recovery. Alterations in serotonin- and dopamine-related responses have previously been discussed in the context of prolonged high-intensity exercise (Meeusen et al. 2006; Roelands and Meeusen 2010). However, peripheral monoamine concentrations provide only indirect insight into central neurotransmission; therefore, their relationship with fatigue-related mechanisms should be interpreted cautiously. Changes in the serotonin-to-dopamine ratio may therefore provide a descriptive indication of peripheral monoaminergic alterations following prolonged maximal rowing exercise. β-Endorphin concentrations increased markedly after exercise and remained elevated during early recovery. Activation of the endogenous opioid system is a well-established response to strenuous endurance exercise and reflects nociceptive modulation and affective regulation under high perceptual load (Boisseau and Delamarche 2000; Bocker and Dishman 2013). Compared with responses reported after shorter rowing tests, the sustained elevation observed here may reflect the prolonged duration and high physiological demands of the 6000-m test (Ostapiuk-Karolczuk et al. 2025; Saanijoki et al. 2018). These findings indicate a pronounced opioid-related response during prolonged maximal rowing.

In contrast, endocannabinoid responses were limited. Anandamide concentrations did not change significantly, while 2-arachidonoylglycerol showed a transient post-exercise decrease followed by recovery. Endocannabinoid responses to exercise are known to be strongly intensity-dependent, with increases more commonly observed during prolonged submaximal exercise than during maximal efforts (Sparling et al. 2003; Raichlen et al. 2013). The present findings are therefore consistent with the high-intensity metabolic profile of the 6000-m rowing test and indicate a less pronounced endocannabinoid response compared with the β-endorphin response under these conditions.

Achievement motivation was assessed before exercise to characterize task engagement during the prolonged maximal effort. Although motivational characteristics have been linked to psychophysiological and hormonal responses in some athletic contexts (Cook and Crewther 2012; Rosa et al. 2020), no associations with acute physiological responses were observed in the present study. This lack of association may be related to the strong physiological demands of prolonged all-out exercise and the relative homogeneity of the elite athlete cohort (McCormick et al. 2015; Brick et al. 2016). These findings suggest that the observed acute physiological responses were more closely associated with the demands of the exercise task than with pre-exercise motivational disposition. Exploratory correlation analysis revealed mainly domain-specific associations among post-exercise biochemical markers. Given the relatively small and homogeneous sample, these findings should be interpreted descriptively rather than as evidence of direct physiological coupling between regulatory systems. Strong relationships were observed between testosterone and the testosterone-to-cortisol ratio, reflecting their mathematical dependence, as well as between serotonin and dopamine concentrations. Similar within-system associations have been reported in studies examining regulatory responses to prolonged and high-intensity exercise (Meeusen et al. 2006; Hackney 2006). In addition, peripheral monoaminergic markers showed positive associations with anandamide, whereas 2-arachidonoylglycerol exhibited no consistent relationships with other variables, consistent with the intensity-dependent nature of endocannabinoid responses to exercise (Raichlen et al. 2013). Therefore, the correlation matrix was retained as a descriptive exploratory analysis and should not be interpreted as evidence of direct physiological interactions between regulatory systems.

From a practical perspective, these findings suggest that early recovery following prolonged maximal rowing may not be fully characterized by traditional endocrine markers alone. Accordingly, the observed regulatory patterns should be interpreted descriptively within the context of a prolonged maximal task, rather than as evidence of direct functional coupling between physiological systems. Future studies may extend this approach by combining endocrine and peripheral neuromodulatory markers with broader metabolic, inflammatory, hematological, catecholaminergic, and muscle damage profiling to provide a more comprehensive characterization of prolonged maximal rowing responses.

## Conclusions

A prolonged maximal 6000-m rowing ergometer test produced distinct temporal patterns of exercise-induced responses across endocrine and peripheral neuromodulatory markers during early recovery. These findings provide a preliminary characterization of peripheral physiological responses observed during early recovery following extended all-out rowing efforts.

### Study limitations

The present study has several limitations. First, peripheral serotonin and dopamine concentrations do not directly reflect central neurotransmitter dynamics, as they may be influenced by peripheral metabolism, platelet storage and release, sympathetic activation, and blood–brain barrier limitations. Therefore, the present findings should be interpreted as peripheral systemic responses associated with prolonged maximal exercise rather than direct evidence of central fatigue mechanisms. Accordingly, the S/D ratio should be considered only as a descriptive index of the relative peripheral monoaminergic response.

Second, the T/C ratio was used as a supportive index of anabolic–catabolic balance and should not be considered a stand-alone marker of metabolic load, endocrine status, or recovery. Third, although the athletes represented a homogeneous elite rowing cohort, formal markers of overtraining or recovery status were not assessed; therefore, possible effects of accumulated training load on baseline hormonal concentrations cannot be excluded. Finally, the study used a focused biochemical profile and did not include broader metabolic, inflammatory, hematological, or hemodynamic assessment. Taken together, the focused biochemical profile, absence of direct fatigue measurements, and lack of broader physiological assessments limit the mechanistic interpretation of the observed responses. Future studies may combine endocrine and peripheral neuromodulatory markers with broader physiological profiling to provide a more comprehensive characterization of prolonged rowing responses.

### Practical applications

The present findings provide preliminary insight into the temporal patterns of endocrine and peripheral neuromodulatory responses during early recovery following prolonged maximal rowing exercise. These observations suggest that the physiological response to prolonged maximal efforts may not be fully characterized by traditional endocrine markers alone.

Future studies combining biochemical markers with performance data, training load, and direct assessments of fatigue and recovery may help determine the potential relevance of these response patterns for athlete monitoring. Confirmation in larger and more diverse cohorts is required before their practical significance can be established.

## Abbreviations

2– AG: 2-Arachidonoylglycerol
AEA: Anandamide
LA: Lactic acid
LMI: Achievement Motivation Inventory
POST: Immediately after exercise
PRE: Before exercise
REC: Recovery
S/D: Serotonin-to-dopamine ratio
T/C: Testosterone-to-cortisol ratio
β–EP: β-Endorphin
